# 556. Using a novel Anaerobic Activity Index to assess risk for poor outcomes in burn patients in a burn intensive care unit

**DOI:** 10.1093/ofid/ofaf695.165

**Published:** 2026-01-11

**Authors:** Natalie A Mackow, Lauren Komarow, Sonia Napravnik, Luther A Bartelt, Lauren DiBiase, Billy J Williams, Felicia Williams, David J Weber, David van Duin

**Affiliations:** Renaissance School of Medicine at Stony Brook University, Stony Brook, NY; George Washington University, Rockville, Maryland; UNC Chapel Hill, Chapel Hill, North Carolina; University of North Carolina School of Medicine, Chapel Hill, NC; UNC Health Care, Chapel Hill, NC; UNC Hospital, Chapel Hill, North Carolina; University of North Carolina at Chapel Hill, Chapel Hill, North Carolina; University of North Carolina, Chapel Hill, NC; University of North Carolina at Chapel Hill, Chapel Hill, North Carolina

## Abstract

**Background:**

Anaerobic antibiotics may be associated with worse patient outcomes in some populations. This has not been studied in burn patients who often receive early antibiotics and are at risk for hospital acquired and multidrug resistant infections.

**Figure 1:**
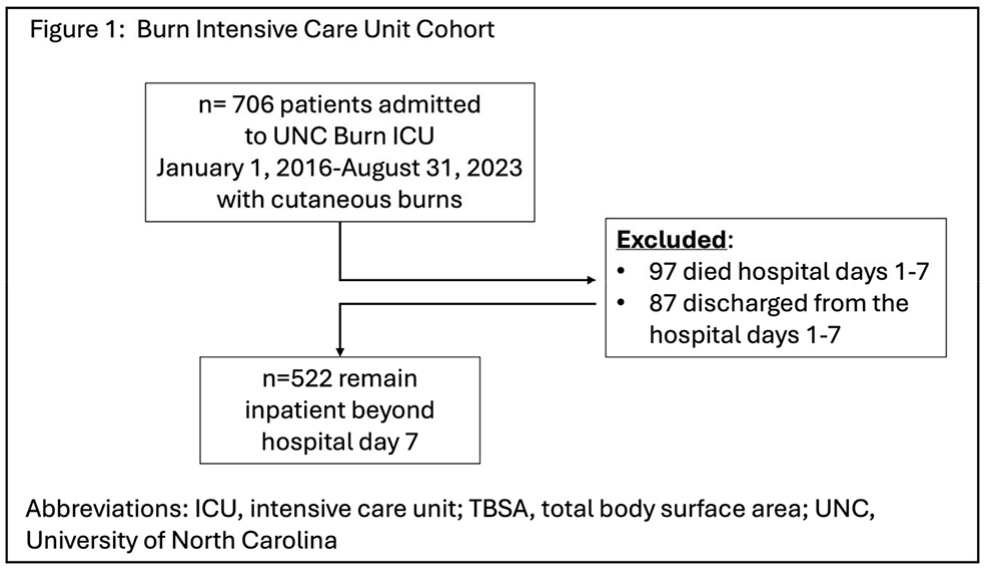
Flowchart of Burn Intensive Care Unit Cohort

**Table 1:**
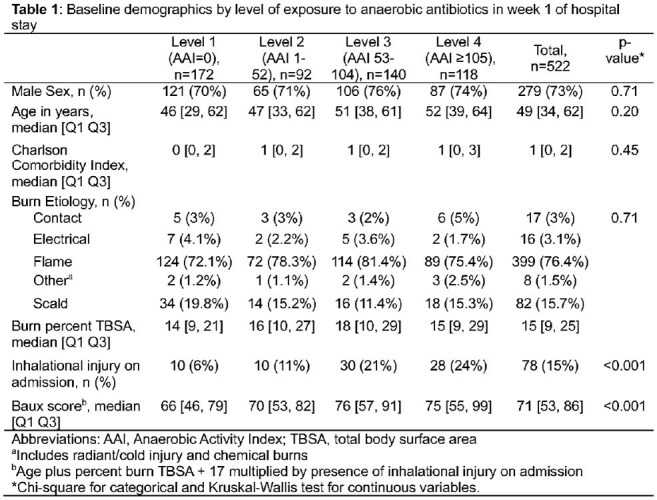
Baseline demographics by level of exposure to anaerobic antibiotics in week 1 of hospital stay

**Methods:**

We performed a retrospective cohort study of adult burn patients admitted to the UNC burn intensive care unit between January 1, 2016, and August 31, 2023. Patients discharged or deceased before hospital day 8 were excluded. Antibiotic exposure was defined as receiving any antibiotic during the first 7-days of hospitalization. A novel Anaerobic Activity Index was created, scoring antibiotics 0-24 based on published in vitro susceptibilities, and multiplied by days on therapy and summed to create a composite anaerobic antibiotic exposure score for week 1 of admission. Anaerobic antibiotic exposure was defined based on quartiles. The primary outcome was a composite 90-day outcome including positive culture for multidrug resistant organisms, healthcare-associated infection, and/or death. Multivariable logistic regression was used to estimate the odds ratio of having the outcome on or after hospital day 8. Two models were generated, adjusting for admission Baux score and Charlson Comorbidity Index.

**Table 2:**
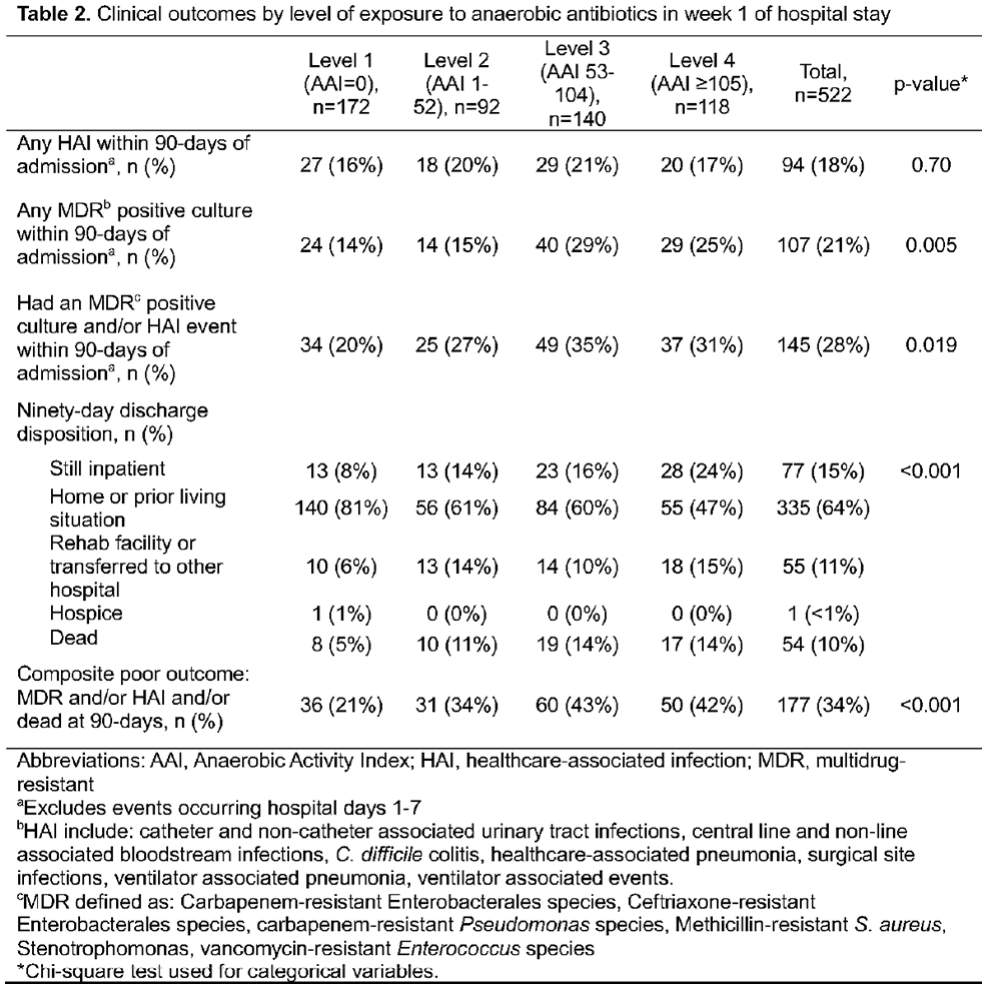
Clinical outcomes by level of exposure to anaerobic antibiotics in week 1 of hospital stay

**Table 3:**
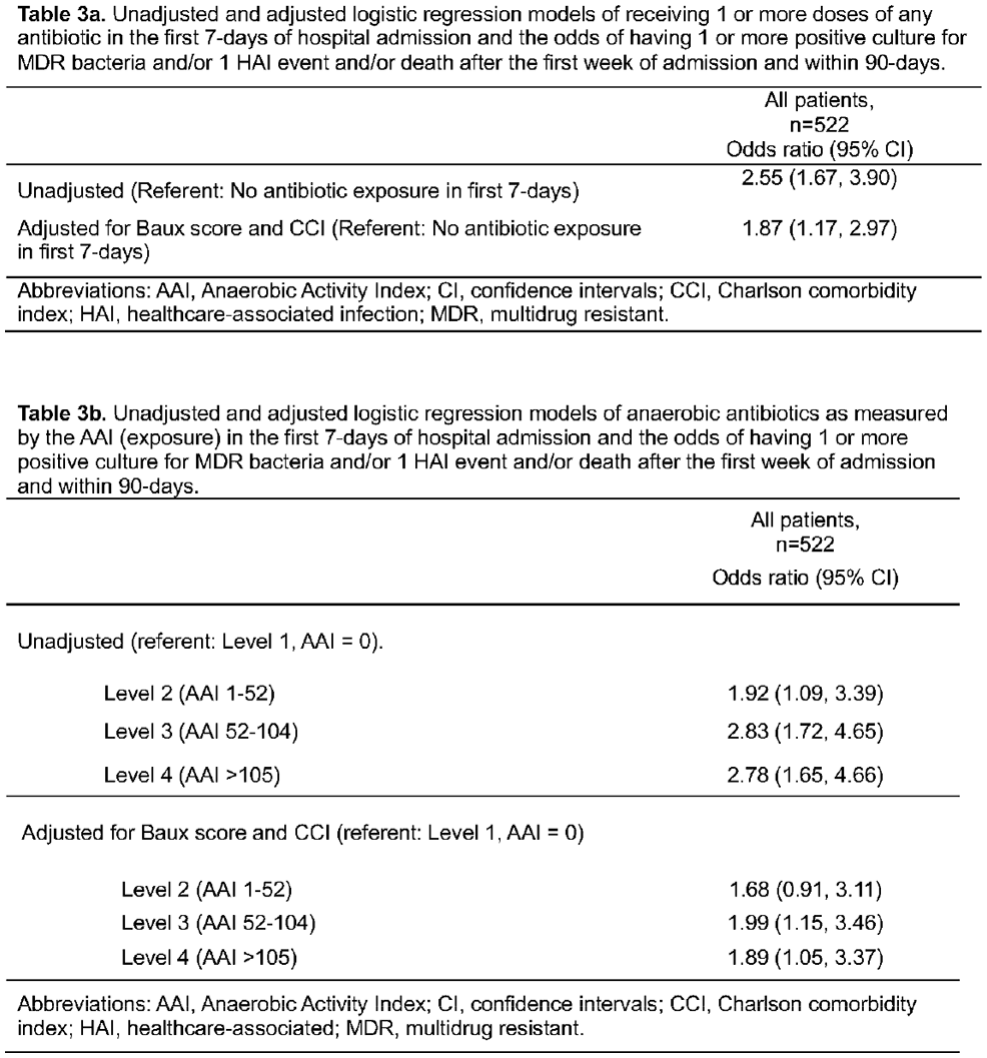
Unadjusted and adjusted logistic regression models of (a) receiving 1 or more doses of any antibiotic in the first 7-days of hospital admission or (b) anaerobic antibiotic exposure as measured by the AAI and the odds of having 1 or more positive culture for MDR bacteria and/or 1 HAI event and/or death after the first week of admission and within 90-days.

**Results:**

In the first week of admission, 67% patients (350/522) received ≥ 1 antibiotic and had 1.87 times the odds of a composite poor outcome compared with those who received no antibiotics in adjusted analyses (95% CI: 1.17, 2.97). The 7-day Anaerobic Activity Index ranged from 0 to 391 with a median of 52 [Q1 Q3: 0, 104]. Patients with level 3 or 4 anaerobic antibiotic exposure had higher baseline severity of illness (Baux score) than those who received less or no anaerobic antibiotics (p < 0.001). Compared to an Anaerobic Activity Index of 0, exposures at levels 3 or 4 were associated with increased odds of worse outcomes (OR [95% CI] 1.99 [1.15, 3.46] and 1.89 [1.05, 3.37], respectively) in adjusted models.

**Conclusion:**

An Anaerobic Activity Index may be used to measure antibiotic exposure over a certain period. In this cohort of critically ill burn patients, early antibiotic exposure was associated with worse outcomes, and increased odds of worse outcomes were seen with higher exposure to antibiotics with activity against anaerobes.

**Disclosures:**

David J. Weber, MD, MPH, CareFusion/BD: Advisor/Consultant|GAMA: Advisor/Consultant|Germitec: Advisor/Consultant|GSK: Advisor/Consultant|Pfizer, Inc.: Advisor/Consultant David van Duin, MD, PhD, AbbVie Inc: Advisor/Consultant|Merck & Co., Inc.: Advisor/Consultant|Merck & Co., Inc.: Grant/Research Support|Parexel International: DSMB|Pfizer, Inc.: Advisor/Consultant|Pfizer, Inc.: Honoraria|Roche Pharmaceuticals: Advisor/Consultant|TEVA: Advisor/Consultant

